# Influence of Ozone Treatment on Ultrafiltration Performance and Nutrient Flow in a Membrane Based Nutrient Recovery Process from Anaerobic Digestate

**DOI:** 10.3390/membranes10040064

**Published:** 2020-04-04

**Authors:** Tobias Gienau, Artjom Ehrmanntraut, Matthias Kraume, Sandra Rosenberger

**Affiliations:** 1 BASF Polyurethanes GmbH, Elastogranstraße 60, 49448 Lemforde, Germany; tobias.gienau@basf.com; 2 Faculty of Engineering and Computer Sciences, Osnabrück University of Applied Sciences, Albrechtstraße 30, 49076 Osnabruck, Germany; artjom.ehrmanntraut@hs-osnabrueck.de; 3 Chemical & Process Engineering, Technische Universität Berlin, Str. des 17. Juni 135, FH 6-1, 10623 Berlin, Germany; matthias.kraume@tu-berlin.de

**Keywords:** nutrient recovery, biological suspension, ultrafiltration, ozone treatment, rheology, membrane performance

## Abstract

Membrane filtration of biological suspensions is frequently limited by fouling. This mechanism is well understood for ultrafiltration of activated sludge in membrane bioreactors. A rather young application of ultrafiltration is the recovery of nutrients from anaerobic digestates, e.g., from agricultural biogas plants. A process chain of solid/liquid separation, ultrafiltration, and reverse osmoses separates the digestate into different products: an organic N-P-fertilizer (solid digestate), a recirculate (UF retentate), a liquid N-K-fertilizer (RO retentate) and water. Despite the preceding particle removal, high crossflow velocities are required in the ultrafiltration step to overcome fouling. This leads to high operation costs of the ultrafiltration step and often limits the economical application of the complete process chain. In this study, under-stoichiometric ozone treatment of the ultrafiltration feed stream is investigated. Ozone treatment reduced the biopolymer concentration and apparent viscosity of different digestate centrates. Permeabilities of centrate treated with ozone were higher than without ozone treatment. In a laboratory test rig and in a pilot plant operated at the site of two full scale biogas plants, ultrafiltration flux could be improved by 50–80% by ozonation. Nutrient concentrations in the fertilizer products were not affected by ozone treatment.

## 1. Introduction

Ultrafiltration (UF) is a state-of-the-art technology for municipal and industrial wastewater treatment, e.g., as membrane bioreactor in combination with activated sludge processes [1]. The interaction mechanisms between biological suspension and membrane surface are well understood and commonly classified into deposition, reversible and irreversible (bio-)fouling, and pore-blocking [1,2]. Solid concentration, particle size and structure as well as sludge rheology have an impact on membrane fouling [3,4]. Many authors link membrane filtration performance to the presence of soluble or macromolecular substances, especially proteins and polysaccharides, which are often summarized as biopolymers, extracellular substances (EPS), or soluble microbial products (SMP) [5,6].

Rather young applications for ultrafiltration of biological suspensions are anaerobic membrane bioreactors [7,8,9] and the recovery of nutrients from manure and anaerobic digestates [10,11]. Anaerobic digestate is a side product of sewage sludge, animal waste, or energy crop fermentation in biogas plants. With 90–95%, it mainly consists of water, but also contains essential plant nutrients like nitrogen (N), phosphorus (P), and potassium (K). Whenever possible, digestates are used as field fertilizer in the vicinity of the biogas plant [12]. Optionally, they can be upgraded into concentrated fertilizer products. This can be an economical option whenever good fertilizing practice otherwise requires transportation and storage of the digestate. After initial solid/liquid separation, e.g., by a decanter centrifuge, membrane processes can be applied for digestate upgrading [13,14]. Ultrafiltration enables the recovery of a retentate and a particle free permeate with dissolved ammonium and potassium. The UF permeate can be further treated in a reverse osmosis (RO) step into concentrated liquid N-K-fertilizer and dischargeable water. Recovery of nitrogen and phosphorus are increasingly important for a sustainable economy, as particularly phosphor is a globally limited resource [15].

The process combination of mechanical solid/liquid separation and membrane treatment is an emerging technology which offers the advantage of producing dischargeable water on the one hand and concentrated, transport worthy nutrient products on the other hand. However, the commercial launch of the process is affected by its high operational costs. Within a process chain of solid/liquid separation, ultrafiltration, and reverse osmosis, the ultrafiltration step has the highest operational energy demand, and consequently the highest operational costs [11,16]. Membrane material, cross flow velocity and transmembrane pressure influence flux performance of the ultrafiltration process, while the flux rates are generally low and limited by organic fouling [10,17]. Fouling mechanisms have been found to be quite similar to those reported for activated sludge filtration, i.e., influenced by particle size concentration, organic macromolecules, and apparent viscosity [13]. Pretreatment and alteration of the ultrafiltration feed stream is thus considered to improve ultrafiltration performance. Successful approaches include precipitation/flocculation [15], thermal treatment [18], and acidification [19].

Ozone treatment of biological suspensions generally results in a disruption of organic compounds. Uzun et al. (2012) investigated the influence of ozone on functional properties of proteins [20], while Wei et al. (2016) observed a dramatic decrease of biopolymer substances in algae suspensions from approximately 50,000 Da to 1000–10,000 Da after ozonation [21]. In wastewater treatment applications, ozone treatment is applied to alter activated sludge properties [22,23,24]. Bougrier et al. (2006) reported an improved anaerobic degradability of ozone-treated activated sludge, while simultaneously rheological sludge properties changed from shear thinning to Newtonian behavior [25]. Sun et al. (2011) showed a 70% reduction of particle size in MBR activated sludge when dosing ozone in a concentration of 500 mg_O3_·g_TOC_^−1^, with positive effects on membrane filtration performance. Ozone dosage of 180 mg_O3_·g_TOC_^−1^ already reduced the membrane fouling rate by 70% [22].

The objective of this paper is to investigate the potential of ozone treatment on the optimization of membrane driven nutrient recovery from anaerobic digestates. As the ultrafiltration is the economically most crucial process step in the nutrient recovery process chain, ozonation is used to alter the fluid properties of the UF feed. Digestates from different biogas plants are treated with varying ozone concentrations and the effect on fluid properties, UF performance, and nutrient split is discussed.

## 2. Materials and Methods

**Figure 1:**
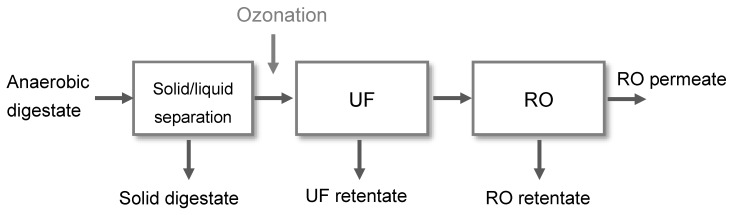
Process scheme of the membrane-based nutrient recovery process.

The membrane-driven nutrient recovery process consists of three process units: solid/liquid separation, ultrafiltration, and reverse osmosis (Figure 1). A detailed description of the process and operational results can be found in Gienau et al. (2018) [11]. The focus of this study is the improvement of the UF filtration performance by ozone treatment of the UF feed (liquid digestate fraction, hereinafter called “centrate”) and its influence on nutrient recovery.

### 2.1. Experimental Set-Up

Digestates were sampled from either digestate storage or post fermenter tanks of different agricultural biogas plants. The samples were further treated and analyzed in the laboratory of UAS Osnabrück. Pilot trials were carried out at the site of two full scale agricultural biogas plants. Table 1 lists significant operational parameters for biogas plant I (BP I) and biogas plant II (BP II). 

All experiments on ultrafiltration performance were carried out with digestate centrate. In the laboratory, solid/liquid separation was achieved by centrifugation at 4300 min^−1^ (3493 g) for 10 min with a laboratory centrifuge Heraeus Megafuge 1.0 (ThermoFisher Scientific, Waltham, MA, USA). For on-site trials, solid/liquid separation was carried out with a screw press separator (agriKomp, Quetschprofi, 3 kW, mesh size: 500 µm) followed by a decanter centrifuge (GEA, Westphalia Separator AD 0509, relative centrifugal force: 3400 g). The on-site solid/liquid separation was improved by cationic polymeric flocculants with a molecular weight of 3 × 10^6^–4 × 10^6^ g∙mol^−1^. The applied concentration of polymers was 4–6 g_Polymer_∙kg_TR_^−1^.

Dry matter content (DM) was 5.8% and 7.7% for BP I and BP II, respectively. For both anaerobic sludges approximately 70% of DM were of organic nature. After solid/liquid separation, a significant reduction of DM and organic dry matter (oDM) was measured. While the digestate contained 5.8–7.7% DM and 4.2–5.5% oDM, the centrate contained 1.9–2.2% DM and 1.1–1.2% oDM.

**Figure 2:**
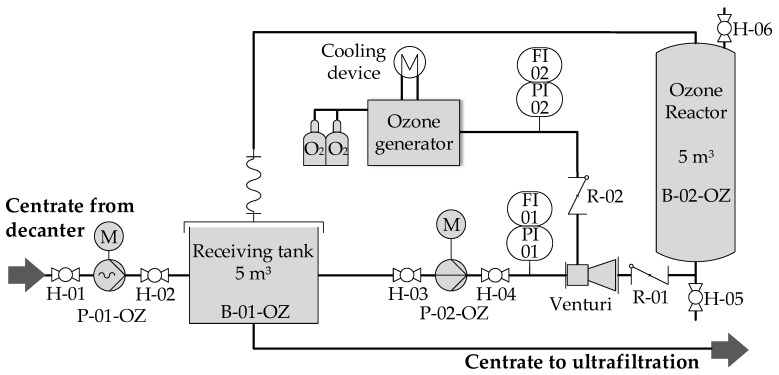
Process flowsheet ozone pilot plant.

Centrate was either directly processed by the ultrafiltration unit or previously treated with ozone. The ozone generator in the laboratory (Sander 301.19) had a performance of 30 g∙h^−1^ and a concentration range of 45–120 g∙m^−3^. For on-site trials, a full-scale ozone generator (Ozonia, CFS-14 2G) with a performance of 980 g∙h^−1^ and a concentration range of 50–150 g∙m^−3^ was used. Both ozone generators were operated with technical oxygen. Slight overpressure (1.5 bars_abs_) was applied. Figure 2 shows the ozone pilot plant (Air Liquide GmbH) with the full-scale ozone generator for on-site pilot trials.

Both ozone installations were operated in batch mode. In the pilot plant, the receiving tank B-02-OZ and the vertical ozone reactor B-02-OZ were initially filled with a total centrate volume of 10 m³. The liquid was then continuously circulated along the ozone venturi injector with a flow rate of 7–8 m^3^·h^−1^ until the desired ozone concentration was reached. Ozone dosages varied depending on the organic fraction in the centrate in a range of 20–150 mg_O3_∙g_oDM_^−1^. Ozone-treated centrate was subsequently stored in a buffer tank.

Ultrafiltration tests in the laboratory were performed with one multi-channel ceramic ultrafiltration module (UF 150, TiO_2_/αAl_2_O_3_, 150 kDa MWCO, Atech, Germany) with 19 channels of 3.3 mm diameter and 1.20 m length. The membrane module had a total membrane surface area of 0.236 m². To understand the influence of operational parameters, the test rig was operated with varying temperatures and crossflow velocities, transmembrane pressure differences of 1.15 ± 0.01 bars, and a recovery rate of 0–48%. 

Permeate flux *J_P_* and recovery rate were calculated by the following equations with permeate flow rate *Q_P_*, feed flow rate *Q_F_* and membrane surface area *A_M_*:

(1) $$J_{P}=\frac{Q_{P}}{A_{M}}$$

(2) $$Recovery=\frac{Q_{P}}{Q_{F}}\cdot 100\%$$

The operational parameters, i.e., crossflow velocity, TMP, and recovery rate of the laboratory UF plant were technically limited within the laboratory installation. Promising results of the laboratory test rigs were thus transferred to pilot scale trials under full scale operating conditions.

The pilot scale UF plant was equipped with 31 multichannel ceramic tubular modules (UF 150, TiO_2_/αAl_2_O_3_, 150 kDa MWCO, Atech, Germany) with a total number of 589 channels of 3.3 mm diameter and 1.20 m length. The membrane module had a total membrane surface area of 7.328 m². During the trials on-site of two full-scale biogas plants, the feed stream entered the UF pilot plant with a temperature of approximately 40 °C, which is the process temperature of the anaerobic biogas process. By using surplus heat of the biogas plant’s CHP unit (combined heat and power), the UF feed could be increased to temperatures around 70 °C. The UF pilot plant was operated at transmembrane pressure differences of 3.5–4.0 bar, crossflow velocities of 4–5 m∙s^−1^, and a recovery rate of 60–70%. 

**Figure 3:**
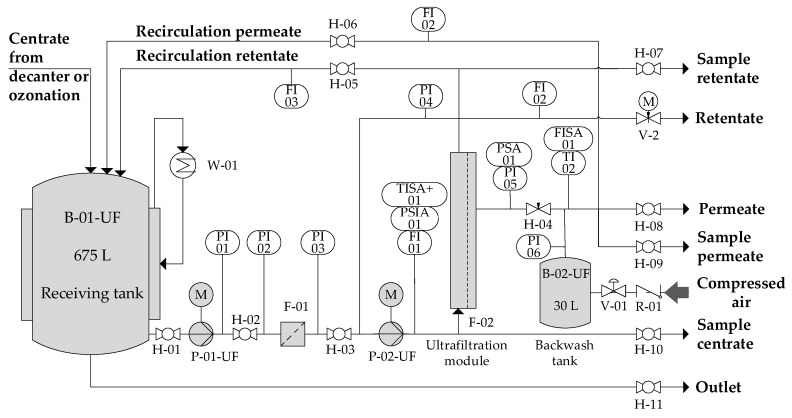
Process flowsheet ultrafiltration pilot plant.

Figure 3 shows the ultrafiltration pilot plant (A3 Water Solutions GmbH). The feed to the ultrafiltration pilot plant came either directly from the decanter centrifuge (reference samples) or from the receiving tank from the ozonation plant (ozone-treated samples). Pilot plant operation and data capture was fully automated. Calculation of permeate flux and recovery was according to Equations (1) and (2). Operation of the UF pilot plant was quasi-continuous—a centrate batch was continuously processed during working hours and operation was stopped during nights and weekends. The UF permeate was further treated in a three-stage reverse osmosis unit.

### 2.2. Analysis and Instrumentation

Dry matter and organic dry matter were analyzed according to the European standards EN 12880 and EN 12879, respectively. The nutrient concentrations were measured with standard Hach vial tests LCK 302 (ammonia), LCK 338 (total nitrogen), LCK 228 (potassium), LCK 350 (phosphorus) in a photometer DR 6000 (Hach). All measurements were carried out as double determination and have a relative error of ≤ 5%. Some nutrient measurements were undertaken by an external agricultural laboratory (AGROLAB GmbH).

Particle size distribution of the samples was determined with a Mastersizer S long bench (Malvern Instruments Ltd., Malvern, UK) at Gent University equipped with a 300 RF lens and an MSX-17 wet sample dispersion unit. 

Size separation and detection of organic fractions of the centrate were performed by Liquid Chromatography–Organic Carbon Detection (LC-OCD, DOC-LABOR Dr. Huber, Karlsruhe, Germany) with a HW-50S column (Tosoh Bioscience). The system consisted of size exclusion chromatography columns for separation of organic molecules according to their molecular size. The separated compounds were detected by UV absorption at 254 nm followed by dissolved organic carbon (DOC) detection. Measurements were carried out by Berlin University of Technology.

The viscosity curve of the liquid phase after centrifugation was measured with a double-gap viscosity system MCR101 (Anton Paar Physica) with the corresponding measuring unit DG 26.7. The viscosity curve was recorded for a shear rate interval of 1–10,000 s^−1^ in a logarithmic ramp of 75 points. Temperature was constant during the measuring procedure.

## 3. Results and Discussion

### 3.1. Influence of Ozone on Fluid Properties

**Figure 4:**
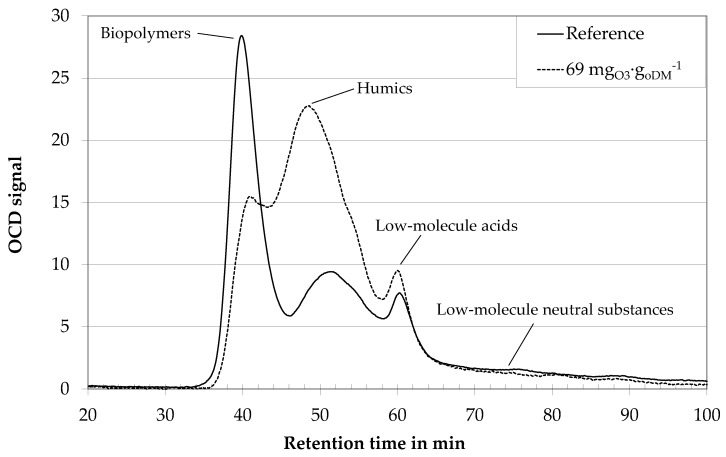
LC OCD analyses of digestate centrate of an agricultural biogas plant with ozone treatment and without. Dilution 1:400.

In a first step, digestate centrates of different biogas plants were treated with ozone dosages of 20–150 mg_O3_∙g_oDM_^−1^. With a mean organic fraction of 20 g_oDM_∙L^−1^ this amounts to ozone concentrations of 0.04–3 g_O3_∙L^−1^. Related to the COD concentration in the centrates of 25–60 g_COD_∙L^−1^, the under-stoichiometric ozone treatment could only result in an oxidation of small fractions of the total organic material. Optically, these low ozone dosages did not result in a decolorization of the generally brown color of the centrates. Nevertheless, ozone treatment changed the fluid properties. Figure 4 gives the LC-OCD analyses of original centrate (reference) and centrate treated with a medium specific ozone dosage of 69 mg_O3_∙g_oDM_^−1^ozone. The first peak of the reference sample arrives at the organic carbon detector at about 38 min after sample injection. It contains biopolymers, i.e., polysaccharides, proteins, and organic colloids. It is followed by a second peak at 50 min, which contains humic substances. The third peak at 61 min is due to the operating conditions (no adjustment of the sample pH and ionic strength of the eluent) and contains organic acids. Concentrations of the different fractions are proportional to areas under the curves. For unknown biological suspensions, it is difficult to distinguish the transitions between the different peaks, though. Therefore, the analysis within this paper is limited to a qualitative analysis instead of a quantification. Research on membrane bioreactors has proven a strong correlation between the biopolymers peak and membrane fouling [5].

Ozone treatment results in a shift of a portion of rather large biopolymers to smaller fractions in the region of the humic substances peak (Figure 4). Under-stoichimetric ozone treatment seems to result in an oxidative disintegration of the long-chained structure of biopolymers, i.e., polysaccharides. This is in good accordance with HPSEC-analyses of an algae suspension, where a reduction of biopolymers (10,000–100,000 Da) towards substances with molecular weights of 1000–10,000 Da was observed [21].

Despite of the shift of biopolymers towards smaller fractions, particle size distribution was not notably affected (data not shown). This is in accordance with literature findings of Erden and Filibeli (2011) [26] and Bougrier et al. (2006) [25] where particle size decrease for ozone concentrations < 50 mg_O3_·g_oDM_^−1^ was within a very small range of 1–2%. On the one hand, even under-stoichiometric ozone treatment seems to destroy part of the biopolymer structure; on the other hand, it might produce more hydrophilic substances, which are enclosed by a hydration shell.

The presence of biopolymers, i.e., polysaccharides in aqueous solutions is known to result in relatively high apparent viscosity levels as well as shear thinning rheological behavior due to the long-chained molecular structure of polysaccharides [27]. Some authors describe an alteration of the rheology of biological suspensions after ozone treatment due to the oxidation of long-chained molecules [22,25].

**Figure 5:**
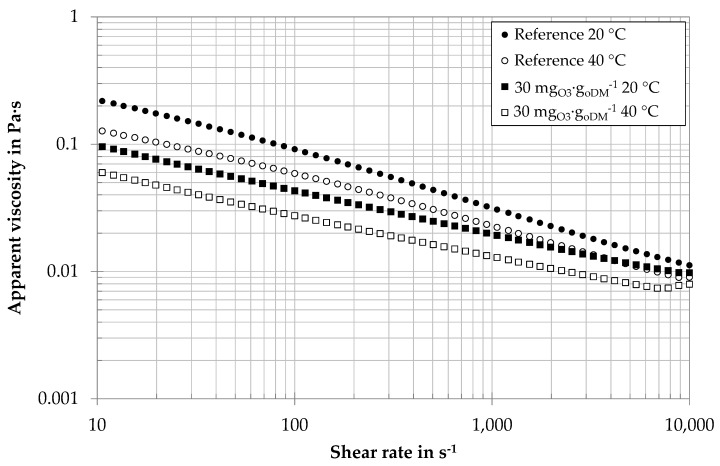
Viscosity curve (each curve average of three different days, Anton Paar MCR 101) of digestate centrate of an agricultural biogas plant with ozone treatment and without (reference).

The influence of ozone treatment was tested for digestates of different biogas plants with specific ozone dosages between 20 and 150 mg_O3_∙g_oDM_^−1^. Even very low specific ozone concentrations of 20–30 mg_O3_∙g_oDM_^−1^ were found to decrease apparent viscosities. For economic and environmental reasons, low ozone dosages of 20 and 30 mg_O3_∙g_oDM_^−1^ were further investigated. Figure 5 shows the example of viscosity curves of a centrate from an agricultural biogas plant with an ozone treatment of 30 mg_O3_∙g_oDM_^−1^ and without ozone treatment. The levels of apparent viscosities are well above water viscosity (η_water_ = 0.001 Pa∙s, 20 °C). All samples are characterized by a shear-thinning rheological behavior: with increasing shear rate, the apparent viscosity of the samples decreases. The increase of the curves for very high shear rates can be explained by the occurrence of Taylor vortices. As the samples are optically free of structural material, both the elevated apparent viscosity level and the pseudoplastic behavior can be explained by the high presence of biopolymers, i.e., the long-chained molecular structure of polysaccharides. For both temperatures, ozone-treated samples show a lower value of apparent viscosity. This is in good accordance with the lower value of biopolymers: the reduction of biopolymer chains reduces the level of apparent viscosity as well as the shear thinning properties.

In a previous screening of 16 different digestate samples, biopolymers as well as centrate viscosity were found to strongly influence the ultrafiltration performance of anaerobic sludge centrates [27]. The described influence of ozone on the centrate is therefore expected to improve the ultrafiltration performance in the described process scheme.

### 3.2. Influence of Ozone on Ultrafiltration Performance

**Figure 6:**
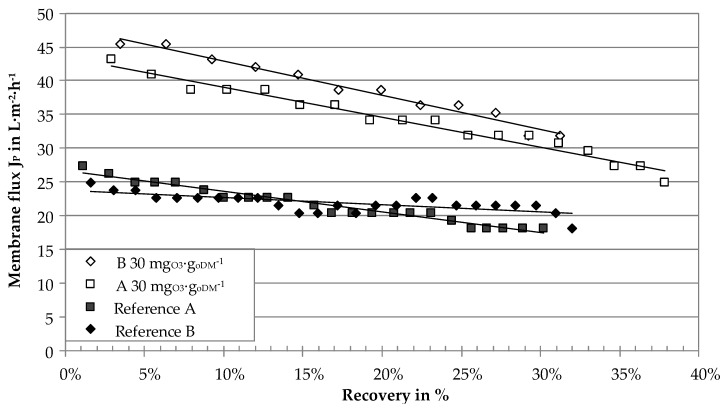
Membrane flux (laboratory ultrafiltration rig) of digestate centrates of two agricultural biogas plants with ozone treatment and without (reference) as a function of recovery, Δ*p* = 1.15 ± 0.01 bar, *v* = 2.46 ± 0.08 m∙s^−1^, *T* = 47.7 ± 1.4 °C

Membrane performance was analyzed as membrane flux *J_P_* of the ceramic ultrafiltration modules in the laboratory test rig and the on-site pilot plant. Figure 6 shows the membrane flux of the ceramic tubular ultrafiltration module measured for two different centrates in the laboratory test rig. Temperature was set to approximately 48 °C (proposed operational temperature in the digestate treatment process chain) and the recovery was continuously increased until a minimum flux rate of approximately 20 L∙m^−2^∙h^−1^ at a TMP of 1.15 bar was reached. This limited the achievable recovery to a maximum value of 38%. Flux rates of the reference samples started at 25–28 L∙m^−2^∙h^−1^ and almost linearly decreased to values of 18–21 L∙m^−2^∙h^−1^. Ozone treatment with 30 mg_O3_∙g_oDM_^−1^ resulted in a significant increase of flux rates with 45 L∙m^−2^∙h^−1^ at low recovery and 30 L∙m^−2^∙h^−1^ at 30% recovery. The difference between the ozone-treated sample and the reference can be expressed in form of a flux enhancement factor according to Equation (3). The flux enhancement by ozone treatment was 1.7 (70%) and 1.8 (80%) for samples A and B, respectively.

(3) $$F=\frac{J_{P,\;Ozone}}{J_{P,\;Reference}}$$

**Figure 7:**
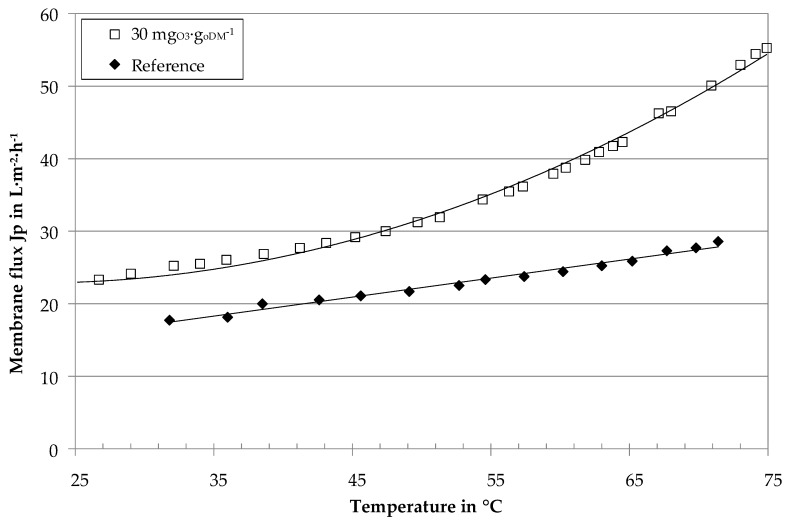
Membrane flux of digestate centrates (pilot plant at BP I) with ozone treatment and without (reference) as a function of temperature, Δ*p* = 4.02 ± 0.05 bar, *v* = 4.6 ± 0.4 m∙s^−1^, 70% recovery.

The pilot plant was quasi-continuously operated with a constant recovery of 70%. Figure 7 gives the membrane flux of BP I at constant recovery and continuously increased temperature. The results confirm the general flux improvement by ozone treatment. The sample with an ozone dosage of 30 mg_O3_∙g_oDM_^−1^ reveals higher flux rates with enhancement factors F of 1.4 at 40 °C, 1.5 at 55 °C and 1.8 at 70 °C. 

The gradient for the ozone-treated sample in Figure 7 increases for temperatures above 45 °C. As a hypothesis, this was explained by a transition from laminar to turbulent flow conditions in the membrane tubes.

Temperature-dependent viscosity can be described by a combination of the power-law equation and an Arrhenius approach to include the temperature dependency of the flow consistency index *k* (see Equation (4) with temperature *T* in *K* and the shear rate $\dot{\gamma }$ in s^−1^).

(4) $$\eta_{n-N}=k\cdot e^{\frac{E_{A}}{R\cdot T}}\cdot {\dot{\gamma }}^{n-1}$$

Table 2 gives the mean values for obtained rheological parameters of agricultural digestate centrate corresponding to the operating conditions of Figure 5 and Figure 6.

Reynolds numbers in pipe flow geometries for non-Newtonian fluids can be calculated according to Equation (5) [13].

(5) $$Re_{n-N}=\frac{{\bar{v}}^{\left(2-n\right)}\cdot d^{n}\cdot \rho }{k\cdot e^{\frac{E_{A}}{R\cdot T}}\cdot {\left(\frac{1+3n}{4n}\right)}^{n}\cdot 8^{\left(n-1\right)}}$$

**Figure 8:**
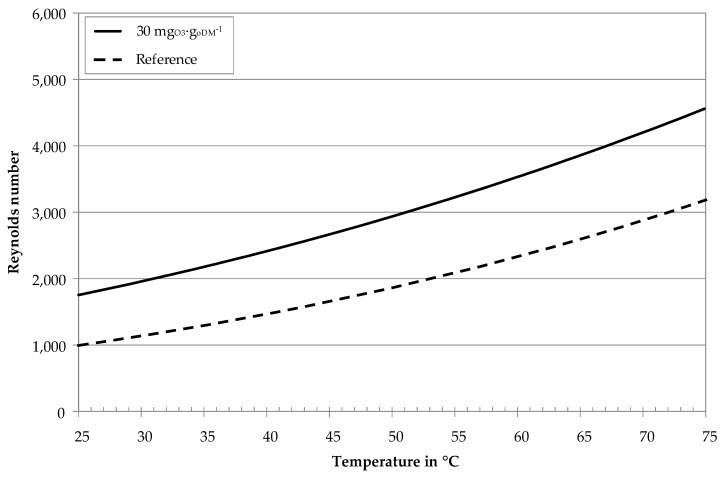
Calculated Reynolds numbers of digestate centrates with ozone treatment and without (reference) as a function of temperature, *v* = 4.6 m∙s^−1^, *d* = 3.3 mm, rheological parameters according to Table 2.

Figure 8 shows calculated Reynolds numbers for rheological parameters of Table 2 and geometry (diameter d = 3.3 m) and flow conditions (v = 4.6 m∙s^−1^) corresponding to Figure 7.

For constant crossflow velocity and geometry, the ozone-treated sample reveals lower viscosity values (Figure 5) and higher Reynolds numbers (Figure 8). The transition from laminar to turbulent flow thus begins at lower temperatures (≈ 40 °C) for the ozone-treated and higher temperatures (≈ 62 °C) for the untreated reference sample. These results undermine the hypothesis regarding the gradient increase in Figure 7. Ozone treatment of digestate centrate thus not only reduces the viscosity of the UF feed, but also leads to a transition towards turbulent flow conditions in the tubular membrane modules. On the one hand, turbulent flow conditions enhance back transportation of particles into the bulk stream. On the other hand, turbulent flow conditions enhance mass transfer phenomena. This leads to a decrease of concentration polarization layer thickness, less accumulation of macro molecules near the membrane surface and accordingly lower cake layer resistance. 

**Figure 9:**
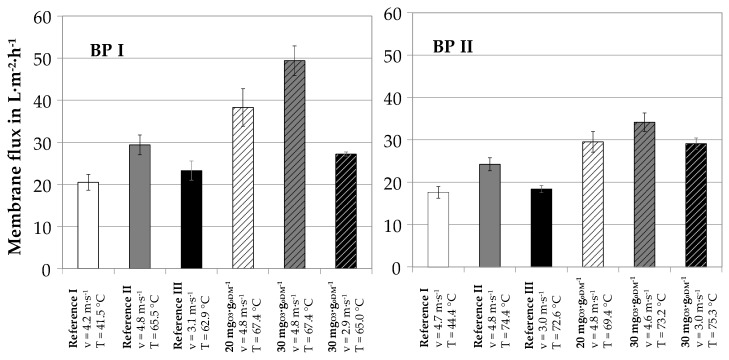
Summary of pilot plant membrane flux values at BP I (**left**) and BP II (**right**), 70% recovery.

Figure 9 shows the accumulated results of flux rates for reference and ozone-treated samples at different operational conditions. The results were obtained with the pilot plant at the site of two different full scale biogasplants (BP I and BP II). 

For mesophilic biogas plants, digestate leaves the biogas plant with a temperature of 40–45 °C. This temperature can typically be maintained in the nutrient recovery process. An increase of centrate temperature to 65–75 °C is possible by heat recuperation if the biogas plant is equipped with a combined heat and power plant and surplus heat is easily available. This was tested at both operational sites. Figure 9 shows that a temperature increase from 45 to 70 °C resulted in mean flux enhancement factors of 1.4 (reference I to reference II). This can be explained by the decrease of apparent viscosity with increasing temperature. In another operational mode, the effect on lower viscosity on Reynold number was used to reduce crossflow velocity. With a mean temperature increase from 45 to 70 °C, crossflow velocity could be reduced from 4–5 m∙s^−1^ to 3 m∙s^−1^ for constant flux rates (reference I to reference III). Ozone treatment of the centrate further increased flux rates: for ozone dosages of 20 mg_O3_∙g_oDM_^−1^, flux enhancement factors of 1.3 and 1.22 (reference II to 20 mg_O3_∙g_oDM_^−1^) were measured at BP I and BP II, respectively. For ozone dosages of 30 mg_O3_∙g_oDM_^−1^, flux enhancement factors increased to 1.68 and 1.4, respectively.

The combination of temperatures increase, ozone treatment and simultaneous decrease of crossflow velocity is an interesting operation mode for full-scale applications. By comparing reference III and ozone dosage of 30 mg_O3_∙g_oDM_^−1^ at crossflow velocities of 3 m∙s^−1^, a further flux enhancement of 1.2 and 1.6 was measured at BP I and BP II, respectively. Compared to the untreated sample, ozone treatment at elevated temperature can result in a considerable reduction of crossflow velocity and parallel flux enhancement (Reference I to 30 mg_O3_∙g_oDM_^−1^ at 3 m∙s^−1^). At both pilot plant operation sites, the described process combination resulted in 50–60% of electric energy reduction for the ultrafiltration process step including ozone generation and treatment. As the ultrafiltration step, i.e., the crossflow pump, has the highest operational energy demand within the total combined process, a 50% reduction of electrical energy demand of the ultrafiltration unit is a considerable step towards economical launch of the membrane-based nutrient recovery from digestates. 

### 3.3. Influence of Ozone on Nutrient Recovery

**Figure 10:**
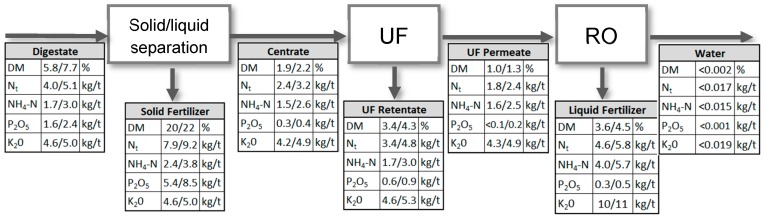
Mean dry matter and nutrient concentrations in process streams of BP I (first value) and BP II (second value).

The complete conditioning process produces four different fractions. The solid fertilizer contains most of particulate organic material, phosphorous and organic nitrogen. The second fraction is the retentate of the ultrafiltration step, which contains all soluble nutrients and some organic substances, e.g., biopolymers. This fraction is usually recirculated to the biogas plant. The third fraction is a particle free concentrated liquid fertilizer, which contains most of the potassium and ammonium. The last fraction is water. Dependent on the RO design, it can be treated to discharge or process water quality. Figure 10 gives the mean dry mass and nutrient concentrations in the different process streams over the whole operation time of the pilot plant at BP I and BP II, respectively. 

**Figure 11:**
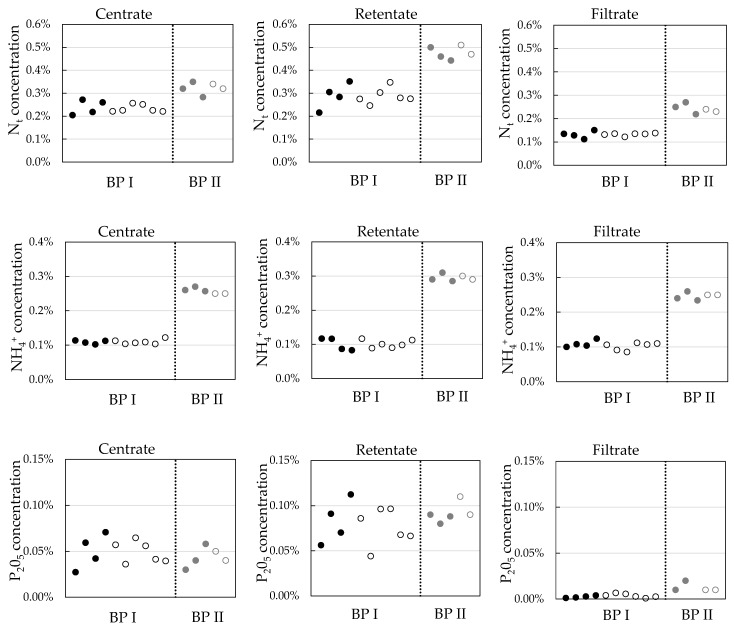
Comparison of total nitrogen, ammonium, and phosphorous concentrations without (full symbols) and with ozone treatment (open symbols) for BP I and BP II.

In the current study, under-stoichiometric ozone treatment is applied to change the centrate properties and improve ultrafiltration performance. As most of the organically bound nutrients are already separated from the liquid stream in the solid/liquid separation step, nutrients in the centrate are mainly salts like ammonium and potassium. Figure 11 gives total nitrogen, ammonium, and phosphorous concentrations in the centrate, UF retentate, and UF permeate with and without ozone treatment. Ozone treatment did not systematically change nutrient concentrations in the process streams.

## 4. Conclusions

The membrane-based nutrient recovery from biogas digestates produces concentrated fertilizer products which allow storage and transportation. The economic application of the process depends on its operational costs, which are dominated by the energy demand of the ultrafiltration step. The presented results indicate that ultrafiltration is essentially influenced by biopolymer concentrations and resulting viscosity in the UF feed, i.e., digestate centrate. Under-stoichiometric ozone treatment with very low ozone dosages can reduce both biopolymer concentration and apparent viscosity. On the one hand, this leads to higher flux rates under otherwise constant operation conditions. On the other hand, the same fluxes as those in the reference case can be achieved with considerably lower crossflow velocities. Ozone treatment prior to ultrafiltration thus offers the potential either to reduce membrane surface area or to reduce operation costs. A negative influence on nutrient recovery was not observed.

## 5. Patents

Based on the described findings, the German patent DE 10 2018 108 450 A1 2019.10.10 “Verfahren und Vorrichtung zur Behandlung einer biologischen Suspension” was disclosed on 10. October 2019.

## Figures and Tables

**Table 1:** Process parameters of biogas plants BP I and BP II.

Process Parameter	Unit	BP I	BP II
Electrical capacity	MW_el_	2.5	1.27
Operational temperature	°C	40–42	39.5–40
Retention time	d	60–65	40–45
Substrate		36% cattle manure 36% maize silage 28% sugar beets	37% slurry/manure 51% maize silage 12% oat/whole plant silage
Digestate storage	m^3^	15,800	13,000
Yearly digestate volume	m^3^∙a^−1^	35,000–55,000	30,000–35,000
Dry matter content digestate	%	5.8 ± 0.4	7.7 ± 0.5
Organic percentage of DM digestate	% of DM	72.8 ± 1.0	71.2 ± 0.3
Dry matter content centrate	%	1.9 ± 0.0	2.2 ± 0.1
Organic percentage of DM centrate	% of DM	59.5 ± 2.7	55.5 ± 2.5

**Table 2:** Mean rheological parameters of digestate centrate with and without ozone treatment.

Parameter	Unit	Reference	30 mg_O3_∙g_oDM_^−1^
k	Pa∙s^n^	$9.3\times 10^{-5}$	$1.9\times 10^{-4}$
n	-	0.63	0.67
E_A_	J∙mol^−1^	21,240	16,770
R	J∙mol^−1^∙K^−1^	8.314	8.314
ρ	kg∙m^−3^	1010	1010
